# Pancreaticopleural Fistula: A Rare Complication of Alcoholic Pancreatitis

**DOI:** 10.7759/cureus.18729

**Published:** 2021-10-12

**Authors:** Malek Ayoub, Janna Ochoa, Daniel Cibich, Mrigank Gupta

**Affiliations:** 1 Internal Medicine, Medical College of Wisconsin, Wauwatosa, USA; 2 Internal Medicine, Medical College of Wisconsin, Milwaukee, USA; 3 Diagnostic Radiology, Medical College of Wisconsin, Milwaukee, USA

**Keywords:** gi stents, pancreaticopleural fistula, pancreatitis, endoscopy, gastroenterology

## Abstract

Pancreaticopleural fistula (PPF) is an uncommon complication of chronic pancreatitis. The authors describe a case of a 41-year-old male with a history of chronic alcoholic pancreatitis and pancreatic pseudocyst who presented with dyspnea and right-sided chest pain for three days. A chest radiograph showed near-complete opacification of the right hemithorax. A diagnostic thoracentesis revealed an exudative, amylase-rich pleural effusion. Endoscopic retrograde cholangiopancreatography (ERCP) demonstrated a normal appearance of the ampulla of Vater and common bile duct; however, there was disruption of the pancreatic duct with leaking beyond the pancreatic neck. A sphincterotomy was performed, and a double-flanged stent was placed, which resulted in the resolution of the dyspnea and the right-sided pleural effusion.

## Introduction

Pancreaticopleural fistula (PPF) has been recognized as a rare complication of chronic pancreatitis. A pleural effusion in a patient with a history of chronic alcoholic pancreatitis should raise clinical suspicion for PPF. The initial analysis of the pleural fluid for amylase will avoid delays in diagnosis. Additionally, nonoperative management using endoscopic retrograde cholangiopancreatography (ERCP) for the stenting of the pancreatic duct is often successful in resolving the fistulous tracts. This would also help reduce significant future morbidity and mortality. This case aims to raise awareness of PPF and aid clinicians in diagnosing and treating this rare diagnosis.

## Case presentation

A 41-year-old male with a past medical history of chronic alcoholic pancreatitis for two years complicated by pancreatic pseudocyst and active tobacco use presented to the emergency department (ED) with progressive shortness of breath and right-sided chest pain that began three days prior. The patient had a total of five episodes of acute pancreatitis in the last year and was diagnosed with a pancreatic pseudocyst three weeks after the remission of his latest episode. Dyspnea was associated with generalized malaise and cough. He denied fever, chills, night sweats, recent travel, or any sick contact. He is an active smoker of 10 pack-years and smokes marijuana recreationally. He started drinking alcohol in his early 20’s and admitted to abusing alcohol. He denied vaping or any other drug use. His medication and family history were unremarkable.

In the ED, he had a blood pressure of 100/60 mmHg, heart rate of 130 beats/minute, temperature of 36.7°C, respiratory rate of 30 breaths/minute, and oxygen saturation of 88% on room air.

On physical examination, the patient was noted to be in a tripod position and uncomfortable with moderate distress. Breath sounds were diminished in the right hemithorax with dullness to percussion. He was also noted to have jugular venous distention and leftward tracheal deviation. Abdominal examination was deferred by the patient due to the severity of his abdominal pain. The rest of the physical examination was unremarkable.

**Figure 1:**
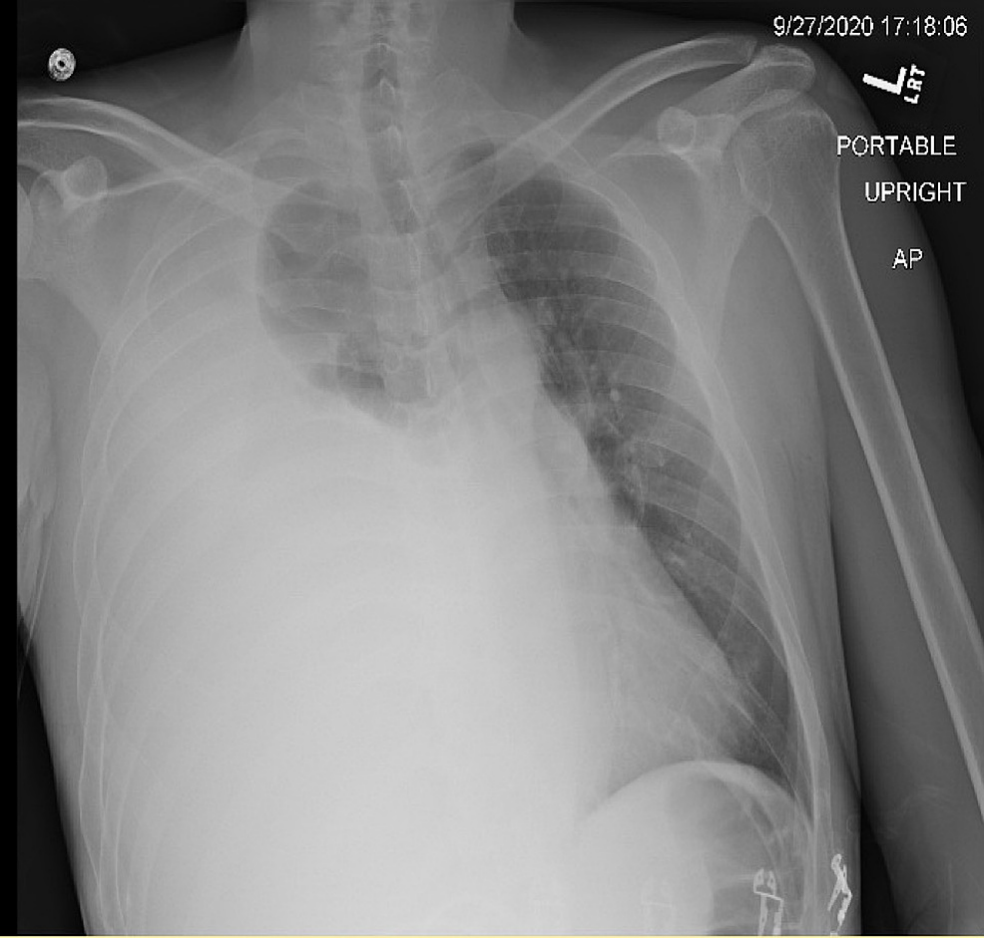
Chest X-ray upon admission Chest X-ray showing opacification of the right hemithorax and a deviated trachea

The initial chest radiograph demonstrated near-complete opacification of the right hemithorax and leftward tracheal deviation (Figure 1). Laboratory examination was significant for a white blood count of 13.1 x 10^9^/L, hemoglobin of 12.4 g/L, and platelets of 427,000 platelets/μL. His serum creatinine was 2.25 g/dL, and his serum lipase was 247 U/L (reference range: 7-60 U/L). The liver function panel was within normal limits (Table 1). 

**Table 1:** Laboratory tests upon admission

Test Results	Values	Reference Ranges
White blood cells	13.1 x 10^9^/L	3.9–11.2 x 10^9^/L
Hemoglobin	12.4 g/L	13.5–17.5 g/dL
Platelets	427,000/μL	150,000–450,000/μL
Creatinine	2.25 g/dL	0.74–1.35 mg/dL
Lipase	247 U/L	7–60 U/L
ALT	42 U/L	7–55 U/L
AST	36 U/L	8–48 U/L
Albumin	4.2 g/dL	3.5–5.0 g/dL

**Figure 2:**
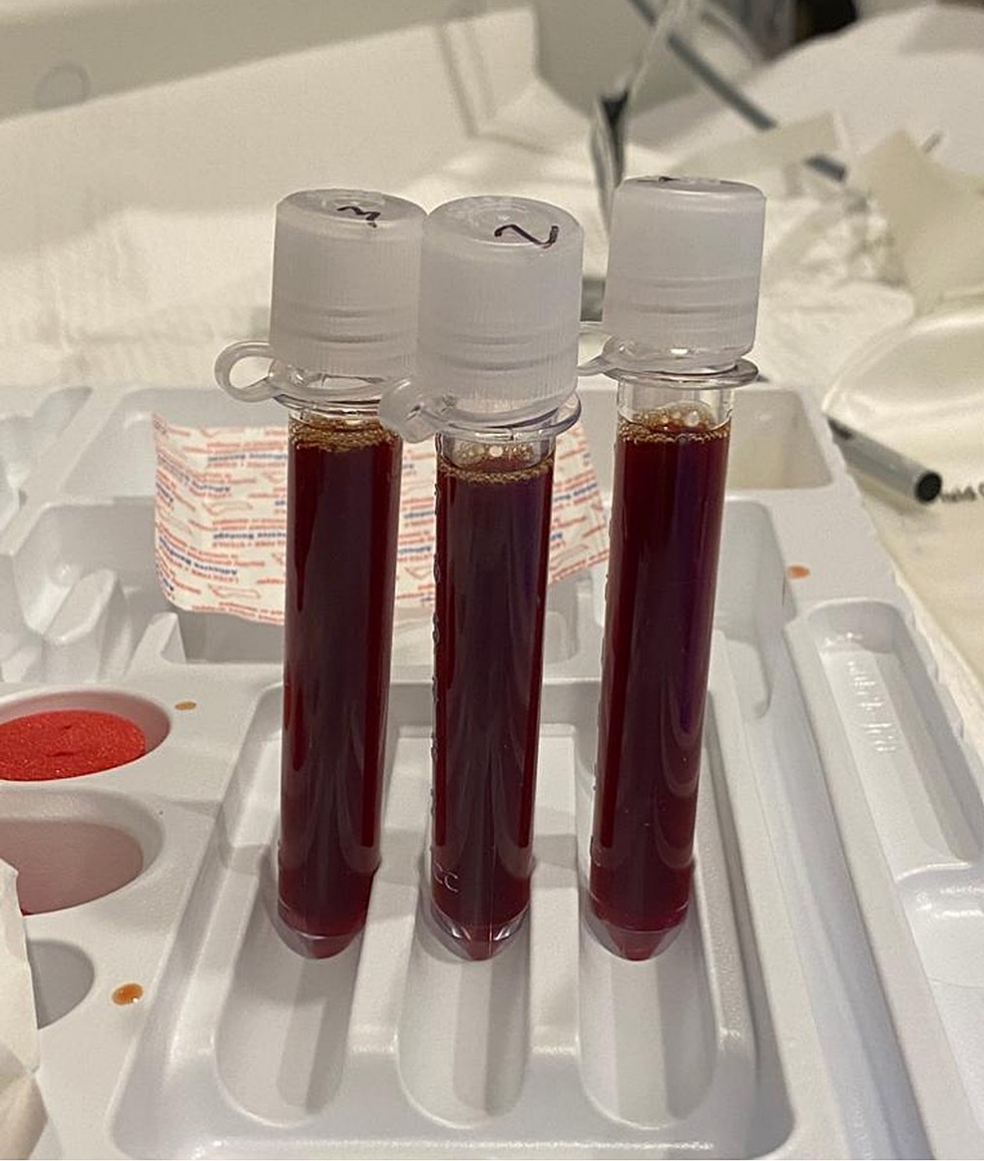
Hemorrhagic pleural fluid

The patient underwent a right thoracentesis with the removal of 1 L of deep red fluid (Figure 2). Pleural fluid analysis revealed 13,465 nucleated cells/mm^3^, 86% polymorphonuclear leukocytes (PMNs), 9% monocytes, 5% pleural lining cells, glucose of 31 mg/dL, pH 7.24, pleural fluid lactate dehydrogenase (LDH) level of 1,031 U/L, serum LDH level of 126 U/L, pleural fluid protein level of 4.4 g/dL, and serum protein level of 6.8 g/dL, consistent with an exudative pleural effusion. Additional testing revealed an elevated pleural amylase of >6554 U/L higher than the laboratory could measure. Fluid cytology, culture, and gram stain were negative (Table 2). 

**Table 2:** Results of diagnostic thoracentesis

Pleural Fluid	Values
Nucleated cells	13,465/mm^3^
Polymorphonuclear leukocytes	86%
Monocytes	9%
Pleural lining cells	5%
Glucose	31 mg/dL
pH	7.24
Lactate dehydrogenase	1,031 U/L
Amylase	>6554 U/L
Serum	Values
Lactate dehydrogenase	126 U/L
Protein	4.4 g/dL

A post-thoracentesis chest radiograph was done within 24 hours and showed rapid reaccumulation of the right pleural effusion. A chest tube was placed for continuous fluid drainage. Subsequently, a computed tomography (CT) of the chest showed resolution of the pleural effusion and bibasilar atelectasis. The patient remained NPO during that time. However, conservative medical management for internal pancreatic fistulae, such as TPN and octreotide, was not done.

A chest tube was placed to drain the reaccumulating pleural fluid, which improved the patient’s shortness of breath. An ERCP was performed, which revealed disruption of the main pancreatic duct with extraluminal contrast extravasation near the pancreatic neck. A sphincterotomy was performed, and a 5-Fr 3-cm double-flanged stent was placed.

The patient was discharged on day five of hospitalization. Prior to discharge, the patient was educated about the possibility of rehospitalization if alcohol use continues and was offered multiple inpatient alcohol rehabilitation options. His pancreatic stent was removed two months later without evidence of a recurrent leak. The patient presented five months after initial admission with another episode of abdominal pain after a relapse of alcohol use. His CT abdomen and pelvis at that time captured the inferior aspect of his thorax, which demonstrated no recurrence of the pleural effusion but did show peripancreatic edema, fat stranding, and mild biliary ductal dilation. The patient was diagnosed with acute pancreatitis without findings suggestive of PPF recurrence.

## Discussion

Pancreaticopleural fistula (PPF) has been recognized as a rare complication of chronic pancreatitis with an estimated incidence of 0.4% [1]. They are most likely associated with alcohol-induced acute or chronic pancreatitis but can also be associated with trauma or iatrogenic injury, among other causes [2]. The pathophysiology of pancreaticopleural fistula development is usually due to a leak from an incompletely formed or ruptured pseudocyst or, in a minority of cases, pancreatic duct leak [3]. Leakage of pancreatic enzymes causes disruption of the fascial layers, forming a fistulous tract between the pancreas and the pleura of the lung. This tract can result in predominately left-sided pleural effusion; however, right-sided and bilateral effusions occur in 19% and 14% of patients, respectively [4]. There is substantial morbidity and mortality associated with PPF, which are largely attributable to sepsis or hemorrhage.

Patients with pancreaticopleural fistulas are often middle-aged men with a history of chronic alcohol abuse and chronic pancreatitis. Patients often present with symptoms of pleural effusions, such as dyspnea, chest pain, cough, fever, and sepsis. Simultaneous pancreatic ascites are present in 20% of the reported cases [5]. Pleural effusions are usually diagnosed with a chest radiograph or CT scan, but identifying the pancreas and fistulous tract as the source of the effusion requires additional workup.

The diagnosis of pancreaticopleural fistula is initially made by performing a thoracentesis with pleural fluid analysis. The pleural fluid analysis would be consistent with an exudative pleural effusion with a high amylase content (normal: <150 IU/L) and high albumin content (normal: >3 g/dL) [2-5]. The differential diagnosis for an exudative fluid with an amylase-rich pleural effusion includes acute pancreatitis, chronic pancreatitis, esophageal rupture, metastatic carcinoma, pneumonia, leukemia, hepatic cirrhosis, and pulmonary tuberculosis [6]. Serum amylase levels vary significantly and are not reliable in diagnosing PPF [7]. A magnetic resonance cholangiopancreatography (MRCP) or ERCP should be performed next to identify whether or not a fistulous tract is present and provide endoscopic treatment.

Magnetic resonance cholangiopancreatography (MRCP) is a noninvasive procedure that does not require contrast administration and is associated with no procedural risks in comparison with ERCP. Although not essential in establishing a diagnosis, MRCP is helpful in diagnosing up to 80% of patients with PPF [8]. It is particularly useful in identifying ductal anatomy, small intrapancreatic and extrapancreatic pseudocysts, or fistulous tracts [9]. ERCP is more invasive and requires sedation; however, it allows for immediate intervention. This includes sphincterotomy and stent placement, which relieves pressure on the pancreatic duct and diverts pancreatic secretions away from the fistula tract, allowing the fistula to close rapidly [10].

Conservative therapies such as total parenteral nutrition and drugs such as octreotide might be utilized to decrease the basal and postprandial pancreatic enzyme secretions. However, these treatments have a success rate of 25%-60% if continued for one month [11]. Schweigert et al. demonstrated that nonoperative managements extending more than 17 weeks were associated with increased risk of complications, such as sepsis and pleural empyema [12].

## Conclusions

Pancreaticopleural fistula is a difficult diagnosis to achieve and to treat at times. Clinicians should have a high suspicion, particularly in the setting of a recurrent unilateral pleural effusion with a history of pancreatitis and alcohol abuse. Initial analysis of the pleural fluid amylase will avoid delay in the diagnosis. Additionally, other options should be considered, such as ERCP with stenting of the pancreatic duct, to prevent significant morbidity and mortality.
